# Associations Between Characteristics of Web-Based Diabetes News and Readers’ Sentiments: Observational Study in the Netherlands

**DOI:** 10.2196/14554

**Published:** 2019-11-13

**Authors:** Hans Vehof, Eibert Heerdink, José Sanders, Enny Das

**Affiliations:** 1 Centre for Language Studies Radboud University Nijmegen Netherlands; 2 Research Group Process Innovations in Pharmaceutical Care HU University of Applied Sciences Utrecht Netherlands; 3 Division Pharmacoepidemiology & Clinical Pharmacology Utrecht Institute for Pharmaceutical Sciences Utrecht Netherlands

**Keywords:** medical journalism, diabetes mellitus, information seeking behaviors, news, diffusion of innovation

## Abstract

**Background:**

Although experts agree that Web-based health information often contains exaggeration and misrepresentation of science, it is not yet known how this information affects the readers’ sentiments.

**Objective:**

This study aimed to investigate whether specific aspects of Web-based diabetes research news are associated with positive or negative sentiments in readers.

**Methods:**

A retrospective observational study of the comments on diabetes research news posted on Facebook pages was conducted as a function of the innovations’ developmental phase, the intended treatment effect, and the use of strong language to intensify the news messages (superlatives). Data for the investigation were drawn from the diabetes research news posted between January 2014 and January 2018 on the two largest Dutch Facebook pages on diabetes and the corresponding reader comments. By manually coding these Facebook user comments, three binary outcome variables were created, reflecting the presence of a positive sentiment, the presence of a negative sentiment, and the presence of a statement expressing hopefulness.

**Results:**

Facebook users made a total of 3710 comments on 173 diabetes research news posts that were eligible for further analysis. Facebook user comments on posts about diabetes prevention (odds ratio [OR] 0.55, 95% CI 0.37-0.84), improved blood glucose regulation (OR 0.68, 95% CI 0.56-0.84), and symptom relief (OR 0.31, 95% CI 0.21-0.44) were associated with less positive sentiments as compared with potential diabetes cures. Furthermore, comments on innovations supported by preclinical evidence in animals were associated with more positive sentiments (OR 1.46, 95% CI 1.07-1.99) and statements expressing hope (OR 1.47, 95% CI 1.01-2.14), when compared with innovations that have evidence from large human trials. This study found no evidence for the associations between language intensification of the news posts and the readers’ sentiments.

**Conclusions:**

Our finding that the attitudes toward diabetes research news on Facebook are most positive when clinical efficacy is not (or not yet) proven in large patient trials suggests that news authors and editors, as well as medical professionals, must exercise caution when acting as a conduit for diabetes research news.

## Introduction

### Background

Patients who monitor online media for health information may experience frequent exposure to exaggeration and misrepresentation of medical science [1-5]. Two typical examples of such infelicitous reporting are the depiction of observed correlations as causal connections—for example, between lifestyle behaviors and disease outcomes—and the inflation of preclinical animal testing results, often followed by the inference of these to humans [6,7]. This is misleading when one considers that about 88% of the pharmaceutical developments that reach the first human trials will never reach the phase of market approval [8]. Such misrepresentations are present in numerous easily accessible health news sites and are spread freely on social media such as Facebook.

Earlier research by our group, on the media coverage of innovative diabetes therapies, found that 83% of Dutch newspaper reports about innovative diabetes treatments lack any reference to clinical trials in humans [9]. Similarly, in the United States, a study on health news appraisals found that most authors do not satisfactorily discuss the quality of the evidence [6].

Although, to our knowledge, there is no literature on the effects of news reporting on the patients’ attitudes, it is highly plausible that messages about promising future treatments could affect the readers’ sentiments such as enthusiasm and curiosity. Moreover, a patient’s level of hope may increase, which is positive as having hope is associated with more favorable diabetes outcomes [10,11]. The effects may also turn out to be negative when, for example, feelings of impatience or disbelief are more prominent.

Overall, 3 aspects of Web-based reporting may influence attitudes. First, the tone of the reports, using intensified language (eg, *revolutionary* and *breakthrough*), a common phenomenon in health news coverage [12], may amplify these sentiments.

Second, attitudes may be affected by the references to an innovation’s developmental phase. Important innovations are covered for many years and during different research stages. For the readers, it may remain unclear as to how long it would take for the innovation to be available in clinical practice. An example is the artificial pancreas, a concept for the treatment of diabetes that has been reported since 1972 [13]. Third, reports on future cure-focused innovations, such as pancreatic cell transplantation for diabetes, may potentially have a stronger impact on the readers’ sentiments than news about noncure-focused treatments.

### Objectives

To increase the understanding of the associations between the characteristics of news about future treatments for chronic illnesses and the readers’ sentiments, we assessed the posts about diabetes research on Facebook pages, together with the corresponding user comments.

## Methods

### Data Source

A retrospective observational study was performed on a corpus of diabetes news messages posted on publicly accessible *Facebook pages* between January 1, 2014, and January 1, 2018, and the associated reader comments. Facebook pages enable public figures and businesses to create a public presence on Facebook. Every person on Facebook can connect with these pages by *liking* them, after which they receive updates in their news feed and can interact with them [14]. The 2 most-followed publicly accessible diabetes pages in the Netherlands were selected: (1) Juvenile Diabetes Research Foundation (JDRF) Nederland [15], the Dutch division of an international type 1 diabetes research foundation, with over 27,000 Facebook followers and (2) Diabetes Fonds [16], a Dutch charity funding of research on all types of diabetes, with over 36,000 Facebook followers.

**Figure 1:**
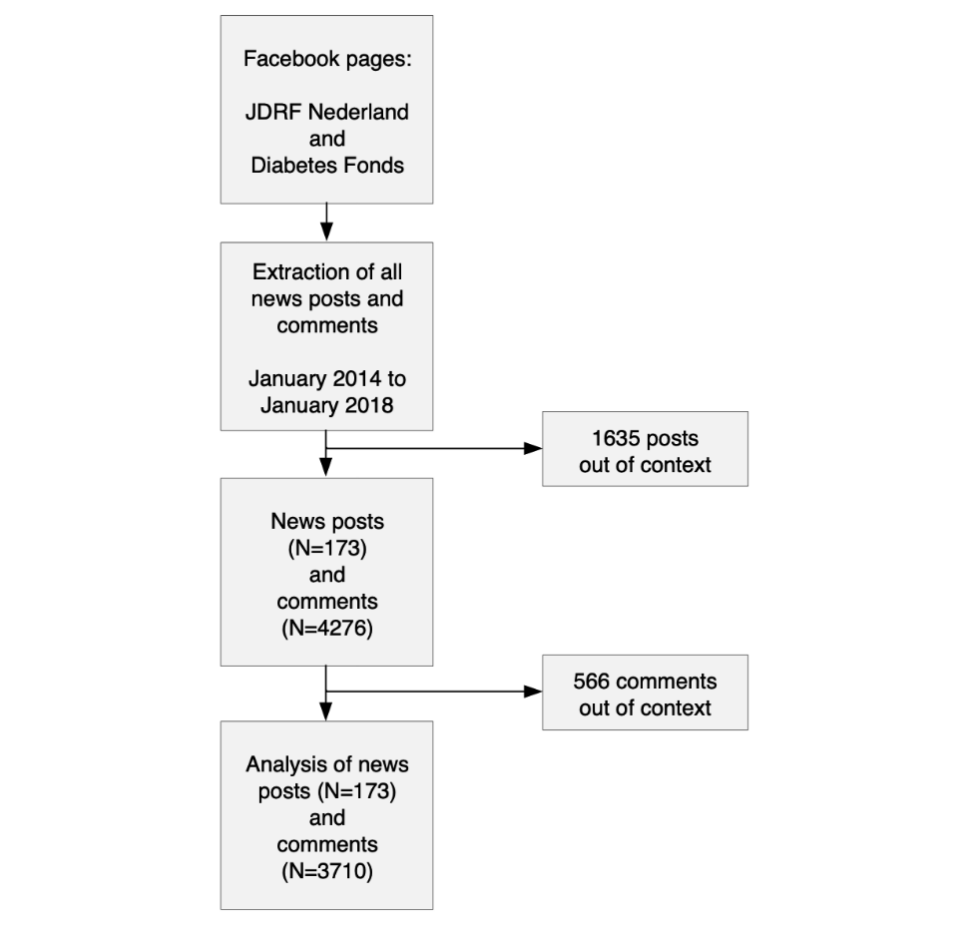
Overview of the data extraction and preparation. JDRF: Juvenile Diabetes Research Foundation.

Data extraction and preparation comprised multiple steps (Figure 1). First, all news posts and associated readers’ comments were extracted for the 4 years from January 1, 2014, to January 1, 2018. The Facepager tool, version 3.8.2., developed by Jünger and Keyling [17], was used to scrape the publicly available data from the Facebook pages, including all reader comments. Replies were excluded (ie, comments on comments) as their content and sentiments are influenced by the initial comments on the news posts. Second, all nonscience news–related posts were identified and removed from the corpus. Furthermore, 3 criteria for diabetes research posts were applied: (1) it must contain information about the development of an innovative therapy, technique, product, instrument, or insights into preventive behaviors; (2) it must contain a reference to a traceable scientist or scientific institution (including medical companies); and (3) it must refer to an innovation which is not (or not yet) applied in the Dutch standard diabetes care. Therefore, nonmedical and nonscientific topics (eg, personal experiences, practical tips, travel stories, and fundraising) were excluded. The consensus between 2 raters in a subsample of 14.99% (271/1808) of the comments on initial Facebook posts was used to resolve disagreements. The third step was to extract the source message (eg, Web-based newspaper item) whenever a hyperlink was available and to merge it with the post content.

### Data Classification

All user comments were evaluated by 2 raters as to whether they contained sentiments (positive, negative, or both). Focus was put on both textual expressions (ie, words and sentences) and the use of emoticons [18,19]. The initial interrater reliability in a subsample of 15% of the sentiments was high (kappa=0.80). For the remainder of the sample, any disagreements were resolved by consensus and, where uncertainties remained, by a third reviewer.

#### Dependent Variables: Positive Sentiments and Written Expression of Hopefulness

In the literature, sentiment analyses generally focus on a combined outcome: *sentiment polarity* (ie, coding a single sentiment expression as either positive, negative, or neutral) [20]. As the readers’ comments may include a combination of positivity (eg, enthusiasm and hopefulness) and negativity (eg, frustrations about waiting for a long time), both positive and negative sentiments were detected and coded for this study.

The binary dependent variable *positive sentiment* in a comment was manually detected and coded as *present*, when the following paraphrase described the utterance correctly: The Facebook user had the aim to express a positive emotion, attitude, or affective state in reaction to a corresponding post about innovative diabetes treatments. Signal examples were expressions of interest, curiosity, enthusiasm, attraction, desire, admiration, surprise, amusement, hope, excitement, gratitude, thankfulness, joy, elation, triumph, jubilation, patience, and contentment [21-23].

Individuals on the Facebook pages on diabetes frequently express that they *have hopes* or *are very hopeful*, or type *let’s hope so!* Hope is a distinct positive sentiment that is considered essential for chronic patients to cope with their disease [24,25]. Having hope can be defined as perceiving a pathway from a negative situation to a favorable state of affairs [26]. Higher levels of hope were found to be associated with a lower prevalence of diabetes [10] and lower mortality in elderly people with diabetes [11].

A binary hopefulness-estimate was created to put a focus on hope as a distinct and essential positive sentiment. In all Facebook comments, the presence of the following Dutch and English conjugations and adjectives based on the verb *to hope* (Dutch: *hopen*) was programmatically detected by using Python programming language [27]: *hoop*, *hoopt*, *hopen*, *hoopte*, *hoopten*, *gehoopt*, *gehoopte*, *hopelijk*, *hope*, *hoped*, *hoping*, *hopeful*, and *hopefully*.

#### Dependent Variables: Negative Sentiments

The binary dependent variable *negative sentiment* in a comment was manually detected and coded as *present*, when the following paraphrase described the utterance correctly: The Facebook commenter had the aim to express a negative emotion, attitude, or affective state in reaction to a corresponding post about innovative diabetes treatments. Signal examples were expressions of indifference, habituation, boredom, aversion, disgust, revulsion, alarm, panic, fear, anxiety, dread, anger, rage, sorrow, grief, frustration, disappointment, discontentment, and restlessness [21-23].

The act of expressing and sharing these Facebook post–related emotions reveals an underlying negative sentiment toward the aspects of the innovation or the news message. Commenters sharing, for example, their boredom or indifference on the Web are unlikely to recommend other patients to try the innovation in the future, nor will they follow the news about the therapy actively; positive and neutral reactions would leave these latter 2 behavior options open.

#### Independent Variables: Intended Therapeutic Effect

Overall, 2 raters identified the intended therapeutic effects of diabetes innovations through discussion and then specified the 5 major categories. These therapeutic effects yielded by diabetes research ranged from simple, practical solutions to a complete cure (Table 1).

**Table 1:** A total of 5 categories of intended diabetes research effects with examples.

Intended effect	Examples
Prevention	Research into a viral trigger for type 1 diabetes; effects of hygiene; nanotechnology; and early diagnosis
Practical solution	Hypoglycemia alarm watch; an insulin temperature sensor; hypoglycemia watchdog; glucose monitoring app; and diabetic shoe
Symptom control and relief	Cognitive behavioral therapy; research on nephropathy; and research on cardiomyopathy in type 2 diabetes
Glucose regulation	Artificial pancreas; inhalable insulin; Cone Insulin G1; and an insulin delivery system
Diabetes cure	Beta cell encapsulation; viral gene transfer; the discovery of immature beta cells; transplanting pancreatic cells; and the effects of vitamin D

#### Independent Variables: Developmental Phase

Furthermore, the developmental phases of innovative diabetes therapies were identified. First, the references to research results in the Facebook posts itself were searched for. When a reference was missing, and a hyperlink was available, the source of the news message was examined. In a previous study, our research group distinguished the different research phases, or the levels of evidence, in health news [9]. Health news articles may contain a reference to positive results from (in the increasing order of reliability) (1) observational, often epidemiological, studies, (2) fundamental research on concepts and theories to improve understanding, (3) preclinical (eg, animal studies) and nonclinical studies to support concrete product development, and increasing the chances for clinical trials in humans to start soon, (4) clinical trials in a small sample of humans (eg, phase II pharmaceutical trials), (5) clinical trials in a large population of humans (eg, phase III pharmaceutical trials), and (6) reports on near-market entry whenever a marketing registration has been or soon will be provided by domestic or overseas authorities. The developmental phase was labeled as *not described*, when the research phase was not recognizable either in the Facebook posts or the source message. In total, 2 raters independently scored a subset of 15% of the news posts (kappa=0.92). Consensus was used to resolve disagreement and indistinctness, and 1 rater subsequently coded the remaining 85%.

#### Independent Variables: Language Intensity

Language intensifiers were defined as words that are used to enhance and give emotional context to the other words that they modify. Literature also refers to such words as the *pars pro toto* superlatives [28,29].

By using Python programming code [27], all words in all Facebook posts were automatically counted and listed in the order of word usage frequency. First, after selecting 2 commonly used designations of the US Food and Drug Administration, *breakthrough* and *promising* [3] (Dutch: doorbraak and veelbelovend), 2 raters discussed and selected the following 15 most frequently used language intensifiers used in the diabetes research news posts: fabulous, beautiful, great, special, important, at last, lifesaving, discovery, dream, positive, powerful, truly, enormously, super, and happy (Dutch: geweldig, mooi, fijn, bijzonder, belangrijk, eindelijk, levensreddend, ontdekking, droom, positief, krachtig, werkelijk, ontzettend, super, and blij). Second, the 17 intensifiers were searched for and counted per Facebook post, and the number was converted into a 3-category variable: *no text intensifiers*, *1 or 2 intensifiers*, and *3 to 9 intensifiers*.

### Analysis

The IBM SPSS Statistics program, version 25, was used to evaluate the differences in the probabilities that sentiments (positive, negative, and hopefulness) were reflected in the user comments, depending on the developmental phase, intended therapeutic effect, and the presence of language intensifiers in the text. Crude and mutually adjusted binary logistic regression models were used to calculate odds ratios (ORs) and 95% CIs. Furthermore, it was assessed whether Facebook pages ID, commenter ID, and gender data contributed to the logistic regression models.

## Results

### Innovative Methods

Table 2 shows that between January 1, 2014, and January 1, 2018, a total of 173 diabetes news messages about innovative methods to treat diabetes were posted on the 2 largest publicly accessible Facebook pages in the Netherlands. These posts evoked 3710 reader comments, containing a total of 2727 positive, 880 negative, and 363 neutral sentiments and 513 verbal expressions of having hope.

**Table 2:** The number of extracted news posts and user comments by the innovations’ intended therapeutic effect and developmental phase, and the number of comment sentiments.

News characteristics and comment sentiments		News posts, n	User comments, n (%)	Comments per post, mean
Total		173	3710 (100)	21
**Intended therapeutic effect**				
	Disease prevention	16	211 (5.69)	13
	Practical solution	14	250 (6.74)	18
	Symptom relief	11	182 (4.91)	17
	Improved glucose regulation	56	1341 (36.15)	24
	Cure	76	1726 (46.52)	23
**Developmental phase**				
	Evidence from fundamental research	47	859 (23.15)	18
	Evidence from pretrial phases	32	758 (20.43)	24
	Evidence from small human trials	33	712 (19.19)	22
	Evidence from large human trials	10	434 (11.70)	43
	Near-market entry	14	259 (6.98)	19
	Evidence from observational studies	12	185 (4.99)	15
	Not described	25	503 (13.56)	20
**Comment sentiments**				
	Positive only	—^a^	2467 (66.50)	—
	Negative only	—	620 (16.71)	—
	Mixed positive and negative	—	260 (7.01)	—
	Positive, including mixed	—	2727 (73.50)	—
	Negative, including mixed	—	880 (23.72)	—
	Neutral	—	363 (9.78)	—
	Verbal expressions of *having hope*	—	513 (13.83)	—

^a^Not applicable.

### Sentiments and the Innovation’s Intended Therapeutic Effect

First, it was tested whether the news messages about the innovative ways to cure diabetes were associated with different sentiments than the news messages related to other therapeutic effects. Table 3 shows that diabetes prevention (OR 0.55, 95% CI 0.37-0.84), improved blood glucose regulation (OR 0.68, 95% CI 0.56-0.84), and symptom relief (OR 0.31, 95% CI 0.21-0.44) were associated with less positive sentiments as compared with potential diabetes cures. Moreover, Table 3 shows that the analyses of negative sentiments show a similar pattern, although this was only significant in blood glucose regulation (OR 1.38, 95% CI 1.12-1.70; for being associated with more negative sentiments). Table 4 shows that, in line with the readers’ positive sentiments, hopefulness was most frequently expressed when Facebook news reported on cure-focused therapies.

**Table 3:** Logistic regression analysis of the association among 3 characteristics of diabetes news in Facebook posts (mutually adjusted) and positive and negative sentiments in the user comments on Facebook pages.

News characteristics		Facebook posts, n	Positive sentiment			Negative sentiment		
			Yes, n (%)	No, n (%)	OR^a^ (95% CI)	Yes, n (%)	No, n (%)	OR (95% CI)
**Intended therapeutic effect**								
	Diabetes prevention	16	94 (3.45)	117 (11.90)	0.55^b^ (0.37-0.84)	85 (9.66)	126 (4)	1.48 (0.98-2.25)
	Practical solutions	14	196 (7.19)	54 (5.49)	1.01 (0.71-1.44)	52 (5.91)	198 (7)	1.01 (0.71-1.45)
	Symptom relief	11	86 (3.15)	96 (9.77)	0.31^b^ (0.21-0.44)	49 (5.57)	133 (5)	1.24 (0.84-1.82)
	Blood glucose regulation	56	970 (35.57)	371 (37.74)	0.68^b^ (0.56-0.84)	343 (38.98)	998 (35)	1.38^b^ (1.12-1.70)
	Diabetes cure	76	1381 (50.64)	345 (35.10)	1.0^c^	351 (39.89)	1375 (49)	1.0^c^
**Developmental phase**								
	Observational evidence	12	61 (2.24)	124 (12.61)	0.31^b^ (0.15-0.66)	79 (8.98)	106 (3.75)	1.88^b^ (1.17-3.02)
	Fundamental evidence	47	610 (22.37)	249 (25.33)	0.71^b^ (0.53-0.95)	238 (27.05)	621 (21.94)	1.22 (0.92-1.63)
	Preclinical evidence	32	639 (23.43)	119 (12.11)	1.46^b^ (1.07-1.99)	112 (12.73)	646 (22.83)	0.55^b^ (0.41-0.76)
	Small trial evidence	33	541 (19.84)	171 (17.40)	1.06 (0.80-1.42)	147 (16.70)	565 (19.96)	0.75^b^ (0.56-1.00)
	Large trial evidence	10	317 (11.62)	117 (11.90)	1.0^c^	115 (13.07)	319 (11.27)	1.0^c^
	Near-market entry	14	175 (6.42)	84 (8.55)	0.73 (0.52-1.04)	70 (7.95)	189 (6.68)	1.39 (0.90-2.14)
	Not mentioned	25	384 (14.08)	119 (12.11)	1.03 (0.76-1.40)	119 (13.52)	384 (13.57)	0.96 (0.71-1.31)
**Language intensifiers^d^**								
	3-9 intensifiers	21	380 (13.93)	156 (15.87)	0.97 (0.75-1.25)	132 (15.00)	404 (14.28)	1.12 (0.86-1.45)
	1-2 intensifiers	55	1120 (41.07)	314 (31.94)	1.13 (0.94-1.35)	334 (37.95)	1100 (38.87)	1.18 (0.99-1.42)
	0 intensifiers	97	1227 (44.99)	513 (52.19)	1.0^c^	414 (47.05)	1326 (46.86)	1.0^c^

^a^OR: odds ratio.

^b^Statistically significant odds ratio.

^c^For reference category, CI is not applicable.

^d^Text intensifiers were a nonsignificant addition to this model but were left in the model to answer study questions.

**Table 4:** Logistic regression analysis of the association among 3 characteristics of diabetes news in Facebook posts (mutually adjusted) and expressed hopefulness (eg, hopefully and I hope) in the user comments on Facebook pages.

News characteristics		News posts, n	Textual expression of hopefulness		
			Yes, n (%)	No, n (%)	OR^a^ (95% CI)
**Potential therapeutic effect**					
	Prevention	16	15 (2.92)	196 (6.13)	0.92 (0.50-1.72)
	Practical solution	14	10 (1.95)	240 (7.51)	0.18^b^ (0.09-0.36)
	Symptom relief	11	4 (0.78)	178 (5.57)	0.13^b^ (0.05-0.36)
	Glucose regulation	56	136 (26.51)	1205 (37.69)	0.49^b^ (0.38-0.64)
	Diabetes cure	76	348 (67.84)	1378 (43.10)	1.0^c^
**Developmental phase**					
	Observational evidence	12	2 (0.39)	183 (5.72)	0.07^b^ (0.02-0.33)
	Fundamental evidence	47	133 (25.93)	726 (22.71)	0.97 (0.65-1.43)
	Preclinical evidence	32	164 (31.97)	594 (18.58)	1.47^b^ (1.01-2.14)
	Small trial evidence	33	96 (18.71)	616 (19.27)	1.26 (0.85-1.87)
	Large trial evidence	10	46 (8.97)	388(12.14)	1.0^c^
	Near-market entry	14	18 (3.51)	241 (7.54)	0.89 (0.49-1.59)
	Not described	25	54 (10.53)	449 (14.04)	0.93 (0.61-1.43)
**Language intensifiers^d^**					
	3-10 intensifiers	21	80 (15.59)	456 (14.26)	1.15 (0.84-1.58)
	1-2 intensifiers	55	242 (47.17)	1192 (37.28)	1.09 (0.87-1.36)
	0 intensifiers	97	191 (37.23)	1549 (48.45)	1.0^c^

^a^OR: odds ratio.

^b^Statistically significant odds ratio.

^c^For reference category, CI is not applicable.

^d^Text intensifiers were a nonsignificant addition to this model but were left in the model to answer the study question.

### Sentiment and the Innovation’s Developmental Phase

Furthermore, it was examined whether the commenters’ sentiments were related to the covered innovations’ developmental phases. Tables 3 and 4 show that, compared with the success in the larger patient trials, evidence from the preclinical phases led to more positive sentiments (OR 1.46, 95% CI 1.07-1.99). Earlier observational (OR 0.31, 95% CI 0.15-0.66) and fundamental findings (OR 0.71, 95% CI 0.53-0.95), however, led to less positive sentiments. Tables 3 and 4 also show that this sentiment pattern was, for the most part, confirmed in the analysis of negative sentiments and the expressions of hopefulness.

### Sentiment and News Message Language Intensification

It was examined whether the intensification of language was associated with sentiments and hopefulness. However, Tables 3 and 4 show that there were no significant relationships between the language intensification of Facebook posts about diabetes research and the sentiments (positive, negative, and hopefulness) of those who reacted to it on Facebook.

### Controlling for Other Variables

To verify the robustness of our findings, additional variables and levels were tested for any effect on our regression analysis. It was found that the Facebook pages ID (JDRF Nederland vs Diabetes Fonds) did not contribute to the regression models. Furthermore, data on the commenters’ gender were available for 71% (2634/3710) of the comments with identifiable sentiment (the first batch of 2 data extractions). Analysis of this sample showed that controlling for gender did not greatly alter the patterns and magnitudes of our results. In addition, the necessity to include commenter ID as a level in our regression model was rejected owing to the flat distribution of comments by the commenters in the same subsample: 80% (1495/1870) of the commenters commented once only.

Our final model only contained the 3 main independent variables: therapeutic effect, developmental phase, and language intensification.

## Discussion

### Principal Findings

In this analysis of 4 years of Facebook posts and comments on diabetes news and user sentiments, posts about potentially curative innovations were associated with more positive general sentiments than the posts not about potential cures, as expected.

However, unexpectedly, innovations supported by evidence from phases just before human clinical trials showed the strongest positive association with improved general sentiments. The observational research results were associated with the most negative general sentiments in the user comments. In addition, and contrary to our expectations, this study found no evidence for the associations between language intensity and the readers’ sentiments.

### Explanation of Findings

A strong positive association was found between cure-focused innovations and positive sentiments. The explanation for this finding is likely to be the absence of cure-focused therapies, to date, for the debilitating disease that diabetes still is. However, negative sentiments may also be provoked by cure-focused innovation, for example, when frustrations about perceived false promises have the upper hand. The strong negative association between the written expression of hopefulness and the news about noncure-focused innovations suggests that the concept of hopefulness only plays a role regarding the desire for a cure.

When looking at the developmental phases, general sentiments were most positively associated with positive results in the preclinical phases closely before human trials. This finding conflicts with the scientific standard that the proof of concept is demonstrated by doing randomized clinical trials. Although it was not assessed by us, an explanation for the preclinical positivity may be the overly optimistic way in which news outlets frequently cover animal studies [30].

Negative associations of the fundamental research with positive sentiments can be explained using construal level theory [31]. First, as the success of therapies in the earliest research stages is far away in time, the patient’s thinking about these innovations becomes more abstract and the consequent anticipation may decrease. Frequently, the medical applicability of very early stage therapies is indeed abstract. A second explanation may be that bad personal experiences, with waiting for other cure-focused innovations, affect the so-called experiential distance (ie, perception of the chance that treatment may become a reality, based on earlier experiences).

Specific research topics may explain the strong association between observational research and the less positive general sentiments. Both disbelief and powerlessness in readers may have arisen from Facebook posts that describe how patients *should have behaved in the past* to prevent their disease. Furthermore, the association between the less positive general sentiments and the news about near-market innovations may be related to fears and frustrations regarding low availability and the doubt on medical insurance coverage.

A possible explanation for the absence of associations between language intensifiers in the news content and the sentiments may have its origin in the characteristics of our target population. Patients and others interested in diabetes seemed to be able to distinguish between the objective content and the subjective use of language. People commenting on the investigated diabetes pages are involved in the burdens of the chronic disease—enduring much and awaiting therapies for many years—and their emotions may not (or may no longer) be as affected by the subjective language.

### Implications of This Study

Previous studies suggest that exaggeration of medical research is a problem [1-7]. Our study shows no evidence that language intensifiers are associated with the sentiments of the online diabetes populations. However, improved positive sentiments were found regarding the preclinical trials—just before evidence from humans—that give reason to suspect an undesired effect of health news exaggeration. The Facebook comments were enthusiastic and full of hope, despite the fact that about 88% of the pharmaceutical developments that reach the first human trials will never reach the phase of market approval [8].

These findings suggest that exaggeration is not limited to language intensification and other verbal inflation of research findings. It is the sheer coverage frequency of specific health research that may lead to positivity and hope, which is not always justified. The mental shortcut *availability heuristic* relies on the immediate examples that come to a person’s mind, possibly putting too much weight on medical information when they read about it frequently [32]. At the same time, the importance of having hope when suffering from a chronic disease must not be underestimated. Hope mediates the relationship between psychological distress and health status and is an essential factor to cope with a disease [24,25].

High-quality health information is increasingly important. Responsible news authors must give context, interpret scientific findings, filter what is important to their target group, and act as an honest and valid conduit, especially as the role of social media is increasing every year [33]. Furthermore, the specific importance of social media to patients must be emphasized. Platforms such as Facebook or Twitter provide tailored information, increase the accessibility of news, and function as social and emotional peer supporters [34-36]. Being well-informed about scientific developments fulfills an essential need for the health information–monitoring patient [37], and it is known that the patients’ subjective well-being also clearly and positively affects health and all-cause mortality [38].

### Strengths and Limitations in Comparison With Other Studies

To our knowledge, our study is the first to quantitatively investigate the associations between the health news characteristics and the sentiments of readers dealing with chronic illnesses.

Moreover, an extra focus was put on the written expressions of being hopeful, enabling the confirmation of general sentiment associations in 1 specific disease-related sentiment. By assessing common language intensifiers, it was possible to differentiate between the objective characteristics of the news posts and a subjective language component. Our large sample size enabled us to mutually adjust the 3 news-related variables. Moreover, the validity of our sentiment outcome increased as offhand comments were observed, written down in an unforced situation. By using a Python regular expression search, the reliability of finding all language intensifiers was high. In addition, the kappa values for rating sentiments and coding message characteristics were high, and coding consensus was achieved regarding occasional discrepancies.

This study does, however, have limitations. First, it is not known what our population’s exact proportion sizes are regarding the patients, their social context, and others who were perhaps only momentarily interested in diabetes and left a comment. Moreover, although the language intensifiers were included, other journalistic language elements, such as emotionalization of news, were not included as a potentially predicting or modifying factor. One final limitation of the study may be the bias that theoretically occurs when readers with either very strong (rejecting) or neutral sentiments refrain from commenting owing to the sentiment itself.

### Conclusions

By observing the news posts and comments on diabetes research on 2 large Dutch Facebook pages, we found that the readers’ sentiments are associated with both the innovations’ developmental phase and the intended therapeutic effect. However, no evidence was found on the association between sentiments and the presence of commonly used language intensifiers in the health news texts.

Our finding that comments on diabetes news on Facebook have the most positive sentiment, and most frequently express hopefulness when clinical efficacy is not yet proven, suggests that the news authors and editors must exercise caution when acting as a conduit for medical research news. More experimental research is necessary, in various populations, to determine a healthy balance between being optimally informed and avoiding having false hope.

## Abbreviations

JDRF: Juvenile Diabetes Research Foundation
OR: odds ratio
